# Factors associated with reporting multiple causes of death

**DOI:** 10.1186/1471-2288-5-4

**Published:** 2005-01-17

**Authors:** Melanie M Wall, Jinzhou Huang, John Oswald, Diane McCullen

**Affiliations:** 1 Division of Biostatistics, University of Minnesota, A460 Mayo Building MMC 303, 420 Delaware Street S.E., Minneapolis, MN 55455, USA; 2 Center for Health Statsitics, Minnesota Department of Health, 717 Delaware Street S.E., Minneapolis, MN 55455, USA

## Abstract

**Background:**

There is analytical potential for multiple cause of death data collected from death certificates. This study examines relationships of multiple causes of death as a function of factors available on the death certificate (demographics of decedent, place of death, type of certifier, disposal method, whether an autopsy was performed, and year of death).

**Methods:**

Data from 326,332 Minnesota death certificates from 1990–1998 are examined. Underlying and non-underlying causes of death are examined (based on record axis codes) as well as demographic and death-related covariates. Associations between covariates and prevalence of multiple causes of death and conditional probability of underlying compared to non-underlying causes of death are examined. The occurrence of ischemic heart disease or diabetes as underlying causes are specifically examined.

**Results:**

Both the probability of multiple causes of death and the proportion of underlying cause compared to non-underlying cause of death are associated with demographic characteristics of the deceased and other non-medical conditions related to filing death certificate such as place of death.

**Conclusions:**

Multiple cause of death data provide a potentially useful way of looking for inaccuracies in reporting of causes of death. Differences across demographics in the proportion of time a cause is selected as underlying compared to non-underlying exist and can potentially provide useful information about the overall impact of causes of death in different populations.

## Background

In their 1986 paper Israel, Rosenberg, and Curtin [1] gave a sort of rallying call for researchers to consider the analytical potential for multiple cause of death data collected by the United States National Center for Health Statistics (NCHS). Beginning with the implementation of the Eighth revision of the ICD in 1968, the NCHS developed and employed several computer systems to automatically select the underlying cause for each death certificate and to produce multiple cause of death data [2]. The resulting multiple cause of death datasets by year are made publically available through the NCHS website.

Acknowledgment of the potential for multiple cause of death data analysis is increasing in other countries as well [3,4]. For example, the Australian Bureau of statistics point out that using multiple cause of death data allows researchers to: more comprehensively understanding and track death due to chronic disease which do not often appear as the underlying cause of death (e.g. Alzheimer's, diabetes, pneumonia), to provide better documentation on multi-morbid associations and the strength of associations between conditions which led to death (for example by examining the frequency of associations between diseases such as diabetes and ischaemic heart disease), and to assist in identifying problems with the process of recording and coding cause of death information [4].

Multiple cause of death data has been used to look at trends in certain diseases, e.g. HIV [5,6] and lung disease [7], but despite its availability, surprisingly few studies have looked at it broadly. Indeed there is no annual standard summary tabulation report of the multiple cause of death data put out by NCHS. This may be due in part to the overwhelming amount of information that arises when combinations of causes of death are considered. There are an enormous number of complex combinations which could be summarized and perhaps it is not clear what tables may be of general interest.

The purpose of this article is to examine some straightforward relationships of multiple causes of death as a function of factors available on the death certificate (demographics of decedent, place of death, type of certifier, disposal method, whether an autopsy was performed, and year of death). Using all death certificates issued by the state of Minnesota between 1990 and 1998 (326,332 deaths), the current study documents the relationship between these factors and the associated frequency of reporting of multiple causes of death as well as the associated frequency that a cause is considered underlying (after data processing) given that it is mentioned on the death certificate. The implication being that differences found are either due to actual differences in causes of death in these groups or due to systematic biases in the reporting of causes of death, or a combination of both. The study will not be able to discern which is the cause but hopes to contribute at the very least by providing an example of the potential relationships which can be examined with the rich multiple cause of death data.

## Methods

### Data source

The data used are from the Minnesota Department of Health Mortality Database and include entries from 326,332 individual death certificates, which represent all deaths occurring in Minnesota during the period of 1990–1998. Record axis codes (those codes which have been completely data processed) are used for all analyses in this paper rather than entity axis codes.

**Figure 1:**
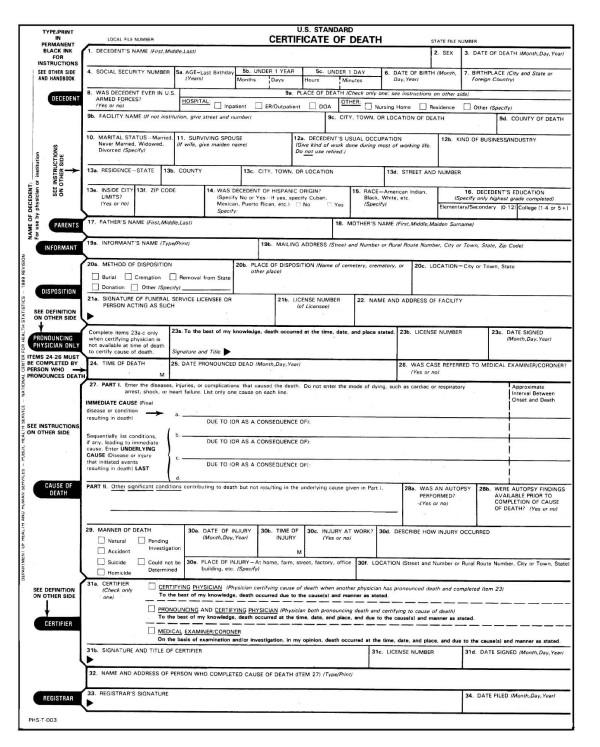
**US standard certificate of death. **Line 27 Part I and Part II are where the causes of death are listed.

**Figure 2:**
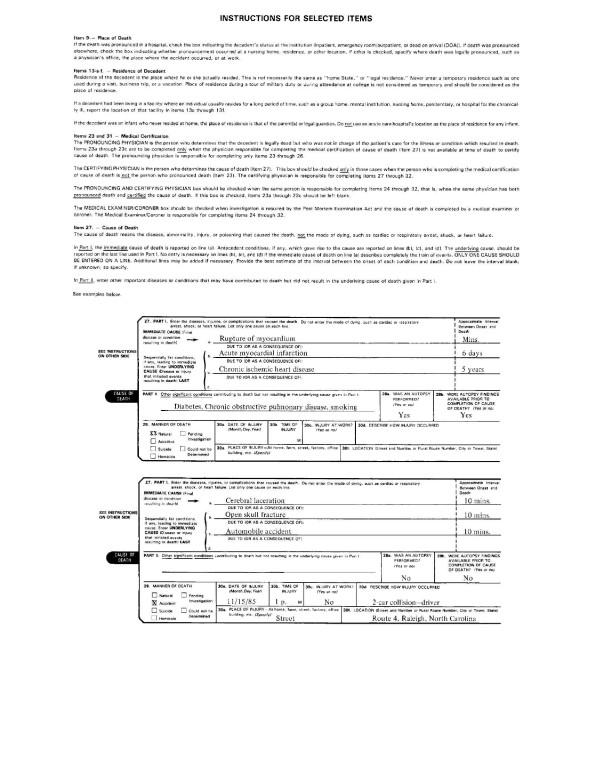
**This figure displays the backside of the certificate of death**. Details are given for filling out specific lines.

A brief description of the entity and record axis coding is given here. The translation of causes of death listed on the death certificate (see Figures 1 and 2 for the actual certificate) to the codes used for statistical analysis goes through many steps. As seen in Figures 1 and 2, the medical information which focuses on the sequence of medical conditions that resulted in death is provided in a two-part format. Part I is for the conditions which directly lead to death, and Part II is for other conditions which contribute to death but are not directly related to the immediate cause of death [1]. The underlying cause of death is defined as the "(a) the disease or injury which initiated the train of events leading directly to death, or (b) the circumstances of the accident or violence which produced the fatal injury" [8]. The entity axis codes represent what is actually written on the death certificate by the certifier expressed in terms of ICD codes including an indicator of which line the code came from and which position on the line it came from (if more than one code was listed per line). While the conditions listed in Part I should form a causal sequence initiated by the underlying cause listed on the lowest line, errors in properly completing the form occur regularly and a reselection of the underlying cause of death is done nationally 30–40% of the time. The decision to reselect an underlying cause other than that listed on the lowest used line in Part I is governed by a set of rules developed by WHO as part of the periodic revision of the International Classification of Disease [9] and is incorporated, along with a complex set of decision tables, into the Automated Classification of Medical Entities (ACME) software. The record axis codes represent a further processing of the entity axis codes to be consistent with the underlying cause data and more amenable to statistical tabulation and analysis. The record axis codes distinguish the ICD code selected as the underlying cause of death and lists all additional causes of death mentioned but does not distinguish them in terms of their ordering or original location on the death certificate. For more detail on entity and record access codes, see [10].

Using the record axis codes, we have for each death record: one underlying cause of death and up to 14 non-underlying causes of death (with no distinction of importance given amongst the non-underlying). When we refer to a cause of death and do not want to distinguish if it is underlying or non-underlying we will refer to it as a "mentioned" cause of death.

In addition to listing one underlying and up to 14 non-underlying causes of death, each death certificate also contains information about the demographics of the deceased, including age, gender, race, marital status, and educational attainment. Also, other conditions related to the death are recorded – place and time of death, who completed the death certificate, if autopsy has been performed and type of body disposal. Minnesota Laws and guidelines govern the process for who and how a death is certified under different circumstances in Minnesota. For example when an unattended death occurs (e.g. at a persons residence) a medical examiner's investigator must arrive at the scene. The medical examiner will contact the last attending physician asking about past medical history of the decedent and most likely cause of death. When an attending physician has seen the decedent within 90 days and the death is natural, jurisdiction is usually given to the physician to certify the death. Sudden or unexpected deaths due in part to any factor other than natural disease must be referred to the medical examiner's office. Autopsies are performed at the discretion of the medical examiner but can also be performed for any death at the request of the immediate family.

The underlying and non-underlying causes of death derived from the death certificate, in this study, are coded according to the 9^th ^revision of International Classification of Disease (ICD). The specific ICD9 codes are grouped into standard reporting of cause of death categories resulting in a total of 107 different causes of death. In this study, individuals are dichotomized as having multiple causes of death (i.e. at least one non-underlying cause) or not having multiple causes of death (i.e. only having an underlying). In addition, because heart disease is the leading cause of death and diabetes is a good example of a disease which often shows up as a non-underlying cause of death, this research investigates two sub -populations: 1) Individuals that have ischemic heart disease (ICD-9: 410 – 414) mentioned as a cause of death (n = 79,833), and 2) Individuals that have diabetes mellitus (ICD-9: 250) mentioned as a cause of death (n = 27,181). For both sub-populations, a dichotomous variable is created to indicate whether the mentioned disease is coded as the underlying cause of death or not.

### Data analysis

Descriptive statistics including total numbers and proportion of all deaths (n = 326,332) in each of the covariate categories are reported as well as proportions of people within each covariate category who have multiple causes of death. In order to examine the association between each covariate and the dichotomous outcome of multiple causes of death, logistic regression is used to mutually adjust each factor for the others. 95% confidence intervals of odds ratios are reported. Trends in multiple cause of death reporting across time are investigated graphically.

Similarly, descriptive statistics including total numbers and proportions will be presented for the two sub-populations with ischemic heart disease (n = 79,833) or diabetes mellitus (n = 27,181) mentioned either as underlying or non-underlying cause of death. Logistic regression is used and 95% confidence intervals are reported to examine factors that are associated with each of these diseases being reported as underlying cause of death rather than non-underlying.

## Results

Overall, 68.9% of the 326,332 deaths from 1990–1998 had at least one non-underlying cause of death in addition to the underlying cause (i.e. have multiple causes). There was a noticeable decreasing trend of reporting multiple causes of death over the 9 year period from 1990 to 1998 with 74.0% in 1990 consistently dropping down to 64.8% in 1996 and remaining around 66% until 1998. Table 1 presents the marginal percentage of individuals in each demographic and death related category as well as the proportions and adjusted odds ratios of having multiple causes of death by each of the covariates. The youngest (<25) and oldest (85+) age groups had the lowest and highest percent of multiple causes of death (61.7% and 71.8%, respectively). Interestingly, the age group from 45–64 did not have higher odds of having multiple causes than the young (<25) group. The percentage of men with multiple causes of death reported was slightly higher (1%) than that of women. Individuals over 25 years old with less education had a higher percentage of multiple causes of death (71.3%) compared to those with higher education (66.6%). The most pronounced difference with respect to demographics was found in race categories, with Native American having the highest percentage of multiple cause of death (74.5%), compared to 68.9% of white.

**Table 1:** Percent of all deaths (n = 326,332) by each covariate. Probability of reporting multiple causes of death given covariate, marginal percent by category, and adjusted odds ratios of reporting multiple causes of death given covariates.

		**% of death by categories**	**% with multiple COD by categories**	**Odds Ratio (95% CI) **^1^
**DEMOGRAPHIC**				
**Age**	**0–24**	3.0	61.7	1
	**25–44**	4.6	69.0	1.38(1.31–1.45)
	**45–64**	13.3	62.1	1.01(0.97,1.06)
	**65–84**	47.8	69.4	1.41(1.35,1.47)
	**85+**	31.3	71.8	1.58(1.51,1.65)
**Sex**	**Female**	50.3	68.6	1
	Male	49.7	69.3	1.11(1.09,1.13)
**Race**	**White**	96.4	68.9	1
	Black	1.7	67.5	1.15(1.078,1.22)
	Native American	0.9	74.5	1.54(1.41,1.69)
	Asian	0.5	65.0	1.04(0.94,1.16)
	Hispanic	0.5	66.6	1.13(1.01,1.26)
**Education^2^**	**High school**	32.4	68.1	1
	Below High School	44.3	71.3	1.03(1.01,1.05)
	Above High School	23.3	66.6	0.94(0.92,0.96)
**Marital Status**	**Married**	41.0	67.4	1
	Single	12.7	68.0	1.08(1.05,1.11)
	Widowed	38.5	71.1	1.09(1.07,1.11)
	Divorced	7.8	68.2	1.12(1.08,1.15)
**DEATH RELATED**				
**Autopsy**	**No**	77.8	69.0	1
	Yes	9.4	74.4	1.57(1.52,1.62)
	Unspecified	12.8	64.7	0.85(0.83,0.87)
**Certifier**	**Physician**	82.0	69.2	1
	Coroner	12.6	70.2	1.23(1.19,1.26)
	Osteopath	1.4	75.3	1.27(1.183,1.37)
	Other/Unknown	4.1	57.8	0.61(0.59,0.63)
**Disposal method**	**Burial**	76.3	70.1	1
	Donation	0.3	69.7	1.02(0.89,1.17)
	Removal	1.5	47.1	0.35(0.33,0.37)
	Cremation	20.5	66.8	0.94(0.93,0.96)
	Unknown	1.4		
**Death place**	**Hospital Inpatient**	35.2	73.4	1
	Residential	19.1	58.7	0.48(0.47,0.49)
	Nursing home	35.5	70.6	0.77(0.76,0.79)
	ER	5.5	65.5	0.64(0.61,0.66)
	Unknown	4.7		

^1 ^Odds ratios are odds of having multiple causes of death given particular category as compared to odds in reference category. Odds ratios are obtained from logistic regression and are mutually adjusted for all other variables.

^2^Education is listed only for population where age>25.

**Figure 3:**
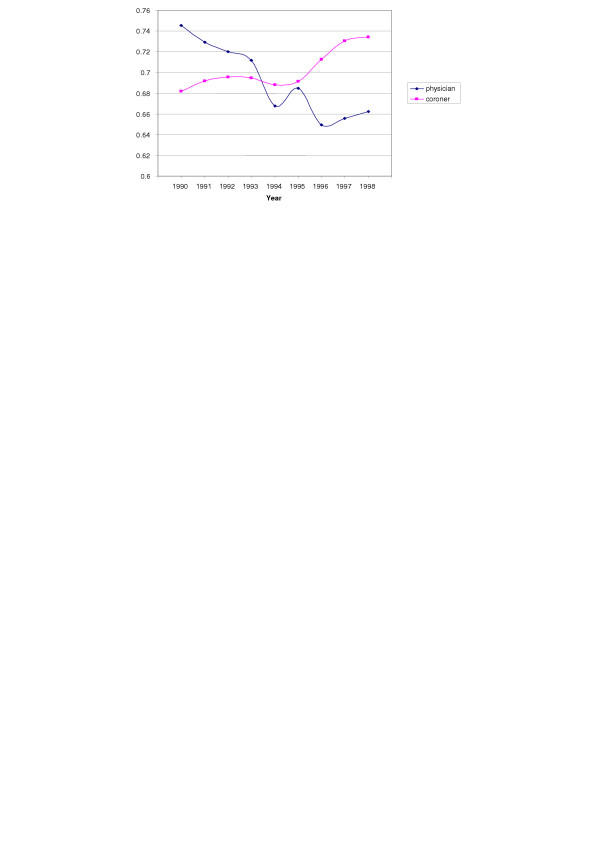
Interaction between certifier and year.

For places of death, hospital in-patient (73.4%) and nursing home (70.6%) had the highest probability of reporting multiple causes of death, and residence had the lowest percentage (58.7%) (Table 1). Between different types of body disposal methods, "removal", which refers to moving the body outside of the US, had the lowest percentage (47.1%) of reporting multiple cause of death. For deaths that had autopsy performed, there was an increased odds of 1.57 that multiple causes of death would be reported. In terms of different types of medical examiners, the difference was less than 1% (69.2% vs. 70.1%) marginally between physician and coroners, the two most frequently seen types of examiners, but examining this difference across time (Figure 3) found an interesting interaction effect in the trend. The physicians showed a decrease in multiple cause of death reporting while the coroners stayed constant or slightly increased over the decade. Table 2 provides reference for the 25 leading underlying causes of death and leading mentioned causes of death in this dataset. It also lists the leading causes of death which occur on death certificates only reporting an underlying cause of death with no non-underlying. The top four causes based on only one cause certificates are the same as the overall top four causes. But it is interesting that the fifth leading cause in this category is "Symptoms and ill-defined conditions" which typically are assigned as the underlying cause only if the sole cause listed.

**Table 2:** Based on Minnesota death records (n = 326,332) from 1990–1998. Top 25 causes of death ranked by underlying and any mention cause of death. Top 25 causes of death for only those deaths where only one cause was listed (i.e. n = 101,423 deaths).

	ranked by underlying cause	number of deaths with underlying cause	% all deaths	rankeded by any mention	number of deaths with cause mentioned	% all deaths	ranked by cause when only one cause mentioned	number of deaths with cause as only one cause	% of deaths with only one cause mentioned
1	Ischemic Heart Disease	61540	0.1886	Other diseases of the Heart	88502	0.2712	Ischemic Heart Disease	13753	0.1356

2	Cerebrovascular Disease	26413	0.0809	Ischemic Heart Disease	79833	0.2446	Other diseases of the Heart	8862	0.0874

3	Other diseases of the Heart	22566	0.0692	Cerebrovascular Disease	43885	0.1345	MN of Trachea, Bronchus & Lung	8157	0.0804

4	MN of Trachea, Bronchus & Lung	18476	0.0566	Symptoms & ill-defined conditions	42196	0.1293	Cerebrovascular Disease	7602	0.0750

5	Pneumonia – except newborn	12465	0.0382	Other mental disorders	38565	0.1182	Symptoms & ill-defined conditions	6459	0.0637

6	Other COPD	11114	0.0341	Pneumonia – except newborn	31312	0.0960	Other mental disorders	3494	0.0345

7	Other mental disorders	10120	0.0310	Diabetes mellitus	27181	0.0833	MN of Breast	3360	0.0331

8	Diabetes mellitus	7959	0.0244	Hypertension without heart disease	26036	0.0798	MN of Intestine, not rectum	3218	0.0317

9	MN of Intestine, not rectum	7388	0.0226	Other COPD	25872	0.0793	Pneumonia – except newborn	3131	0.0309

10	Diseases of the arteries, veins & pulmonary circulation	7181	0.0220	MN of Trachea, Bronchus & Lung	19896	0.0610	MN of Other & unspecified sites	3042	0.0300

11	MN of Breast	6646	0.0204	MN of Other & unspecified sites	19386	0.0594	MN of Prostate	2380	0.0235

12	Symptoms & ill-defined conditions	6488	0.0199	Diseases of the arteries, veins & pulmonary circulation	19180	0.0588	Other COPD	2332	0.0230

13	Transportation accidents – Motor Vehicle	5809	0.0178	Other diseases of the digestive system	17943	0.0550	MN of Pancreas	2313	0.0228

14	Other diseases of the digestive system	5777	0.0177	Pneumoconiosis & other resp. Diseases	17728	0.0543	Diseases of arteries, veins & pulmonary circulation	1758	0.0173

15	Other disease of the Nervous System and Sense Organs	5714	0.0175	Other disease of the Nervous System & Sense Organs	16799	0.0515	Other Neoplasms of lymphatic & Hematopoietic tissue	1701	0.0168

16	MN of Prostate	5669	0.0174	Chronic and Unspec. Nephritis & renal failure & renal sclerosis	16065	0.0492	Alzeimer Disease	1536	0.0151

17	MN of Other & unspecified sites	5504	0.0169	Arteriosclerosis	13879	0.0425	Other disease of the Nervous System & Sense Organs	1477	0.0146

18	Suicide	4435	0.0136	Transportation accidents – Motor Vehicle	10367	0.0318	Residual, Undefined	1300	0.0128

19	MN of Pancreas	4136	0.0127	Medical complications & misadventures	10299	0.0316	MN of Brain, other nervous	1292	0.0127

20	Residual, Undefined	4135	0.0127	MN of Intestine, not rectum	9116	0.0279	Leukemia, & Aleukemia	1271	0.0125

21	Pneumoconiosis & other resp. Diseases	4028	0.0123	Septicemia	8790	0.0269	Perinatal conditions	1206	0.0119

22	Accidental falls	4010	0.0123	Suicide	8740	0.0268	MN of Ovary, Fallopian tube, Broad ligament	1124	0.0111

23	Alzeimer Disease	3757	0.0115	MN of Breast	8704	0.0267	Other diseases of the digestive system	1079	0.0106

24	Other Neoplasms of lymphatic & Hematopoietic tissue	3584	0.0110	Other genito-urinary disease	8382	0.0257	Suicide	990	0.0098

25	Leukemia, & Aleukemia	3440	0.0105	MN of Prostate	8369	0.0256	MN of Kidney	955	0.0094

The results presented so far explored how covariates may be correlated with multiple causes of death being reported. The following results pertain to the conditional probability that a particular cause of death (ischemic hear disease or diabetes) is selected as underlying given that it is mentioned. Results for the subpopulations with ischemic heart disease or diabetes mentioned are shown in Table 3 and Table 4, respectively.

**Table 3:** Population with Ischemic Heart Disease mentioned on death certificate (N = 79833). Marginal percent by category, conditional percent with ischemic heart disease as underlying given that it is mentioned by category and odds ratios of ischemic heart disease being reported as underlying cause when it is mentioned given covariates.

		**% of deaths by category**	**% with heart disease as underlying**	**Odds Ratio (95% CI)**^1^
**DEMOGRAPHIC**				
**Age**	**0–44**	1.7	80.0	**1**
	**45–64**	12.4	81.8	1.2 (1.03,1.36)
	**65–84**	53.0	76.1	0.84 (0.74,0.95)
	**85+**	32.8	76.8	0.87 (0.77,1.00)
**Sex**	**Female**	45.2	76.3	**1**
	Male	54.9	77.8	0.95(0.91,0.99)
**Race**	**White**	97.9	77.2	**1**
	Black	0.8	71.8	0.78(0.66,0.94)
	Native American	0.7	69.7	0.55(0.45,0.66)
	Asian	0.3	77.2	1.12(0.81,1.56)
	Hispanic	0.3	72.2	0.79(0.59,1.06)
**Education^2^**	**High school**	29.1	76.3	**1**
	Below High School	49.1	77.9	1.07(1.03,1.11)
	Above High School	21.8	76.5	1.02(0.97,1.07)
**Marital**	**Married**	44.9	77.5	**1**
	Single	8.2	79.7	1.25(1.16,1.33)
	Widowed	40.0	75.9	1.04(0.99,1.09)
	Divorced	6.9	77.9	1.13(1.05,1.22)
**DEATH RELATED**				
**Autopsy**	**NO**	77.8	76.9	**1**
	Yes	10.5	78.0	0.91(0.86,0.97)
	Unspecified	11.7	77.8	1.12(1.06,1.18)
**Certifier**	**Physician**	79.5	75.5	**1**
	Coroner	15.2	84.3	1.34(1.25,1.42)
	Osteopath	1.5	80.9	1.25(1.07,1.46)
	Other/Unknown	3.8	81.0	1.23(1.13,1.35)
**Disposal method**	**Burial**	78.9	77.3	**1**
	Donation	0.3	75.3	0.74(0.56,0.98)
	Removal	1.6	85.3	1.74(1.48,2.05)
	Cremation	18.0	66.8	0.95(0.91,0.99)
	Unknown	1.2		
**Death place**	**Hospital Inpatient**	36.1	72.9	**1**
	Residential	20.1	90.5	1.83(1.74,1.93)
	Nursing home	27.9	71.2	0.83(0.79,0.87)
	ER	11.4	83.2	1.67(1.39,1.97)
	Unknown	4.5		

^1 ^Odds ratios are odds of having ischemic heart disease as underlying rather than contributing given particular category as compared to odds in reference category. Odds ratios are obtained from logistic regression and are mutually adjusted for all other variables.

^2^Education is listed only for population where age>25.

**Table 4:** Population with Diabetes mentioned on death certificate (N = 27181). Marginal percent by category, conditional percent with diabetes as underlying given that it is mentioned and odds ratios of diabetes being reported as underlying cause when it is mentioned given covariates.

		**% of deaths by category**	**% with diabetes as underlying given that it was mentioned**	**Odds Ratio (95% CI)^1^**
**DEMOGRAPHIC**				
**Age**	0–44	2.3	51.7	**1**
	45–64	13.2	34.5	0.51(0.43,0.60)
	65–84	59.5	27.7	0.37(0.32,0.43)
	85+	24.9	28.4	0.39(0.33,0.45)
**Sex**	**Female**	51.7	30.2	**1**
	Male	48.3	28.3	0.92(0.86,0.97)
**Race**	**White**	95.4	28.9	**1**
	Black	2.0	36.5	1.19(0.98,1.43)
	Native American	1.5	41.0	1.42(1.136,1.76)
	Asian	0.5	27.2	0.88(0.59,1.30)
	Hispanic	0.7	36.6	1.33(0.978,1.82)
**Education^2^**	**High school**	31.2	30.1	**1**
	Below High School	47.8	28.0	0.99(0.93,1.06)
	Above High School	21.0	30.8	1.03(0.96,1.11)
**Marital**	**Married**	43.6	27.8	**1**
	Single	8.5	34.3	1.25(1.13,1.38)
	Widowed	40.2	29.0	1.09(1.01,1.16)
	Divorced	7.7	33.5	1.11(0.99,1.23)
**DEATH RELATED**				
**Autopsy**	**No**	83.3	28.3	**1**
	Yes	5.5	22.8	0.70(0.62,0.80)
	Unspecified	11.3	39.4	1.49(1.37,1.62)
**Certifier**	**Physician**	85.0	29.7	**1**
	Coroner	10.0	24.2	0.69(0.62,0.77)
	Osteopath	1.5	30.4	1.03(0.81,1.29)
	Other/Unknown	3.5	34.3	1.23(1.08,1.42)
**Disposal method**	**Burial**	79.2	28.6	**1**
	Donation	0.3	25.0	0.84(0.51,1.39)
	Removal	1.1	37.0	1.46(1.143,1.87)
	Cremation	18.3	31.4	1.06(0.99,1.14)
	Unknown	1.1		
**Death place**	**Hospital Inpatient**	34.8	25.2	**1**
	Residential	18.8	30.9	1.29(1.19,1.41)
	Nursing home	37.4	32.7	1.57(1.47,1.68)
	ER	6.4	28.3	1.15(1.02,1.29)
	Unknown	2.6		

^1 ^Odds ratios are odds of having diabetes as underlying rather than contributing given particular category as compared to odds in reference category. Odds ratios are obtained from logistic regression and are mutually adjusted for all other variables.

^2^Education is listed only for population where age>25.

Table 3 gives the odds ratio of ischemic heart disease being selected as underlying cause of death when it was mentioned as a cause, given the covariates main effect. Overall 77.1% of the time that heart disease was mentioned as a cause, it was selected as the underlying cause of death. The 45–65 year age group had the highest probability of heart disease being codes as underlying when it was mentioned (81.8%). Males had a slightly lower probability than females to have heart disease as underlying cause of death when it was mentioned on the death certificate. Furthermore, Blacks and Native Americans were less likely to have heart disease coded as underlying cause of death when it was present on the certificate as compared to Whites. Individuals that had an autopsy performed were less likely (0.91 odds ratio) to have ischemic heart disease selected as underlying when it was mentioned. If a physician is the death certifier, the probability of selecting heart disease as underlying cause of death is relatively the lowest when compared to coroner, osteopath and other and unknown certifiers. Amongst body disposal methods, the probability for heart disease to be reported as underlying cause was the lowest if bodies were donated (OR = .7 with "burial" as baseline category), and highest if bodies were removed (OR = 1.7). Finally, for those individuals who had heart disease mentioned on their death certificate, patients who died at a residence (not a nursing home) were most likely to have ischemic heart disease selected as the underlying cause of death (90.5% or an OR= 1.8 compare to hospital in-patient).

Unlike Ischemic heart disease, only 29.3% of deaths with diabetes mentioned on the certificate had it selected as the underlying cause of death. While only 2.3% of deaths with diabetes mentioned occurred in the youngest age group (0–44 years), (Table 4) this group has a much larger probability of having diabetes be the underlying cause compared to non-underlying (51.7% reported as underlying). Men were less likely to have diabetes selected as underlying when it is mentioned on the certificate than women. Blacks and Native Americans both have significantly higher odds (OR = 1.2 and 1.4, respectively) of diabetes being the underlying cause given that it was mentioned as compared to Whites. The role of autopsy is that it was less likely diabetes was reported as underlying (OR = 0.7) when one was performed than if one was not. Moreover, if a coroner was the death certifier, diabetes was less likely to be reported as underlying. An increase in the reporting of diabetes as underlying was found for deaths that were removed. Finally, deaths occurring outside of the hospital inpatient setting all show increased odds of diabetes being selected as the underlying cause of death when it has been mentioned.

We also examined what other leading causes of death showed up as underlying when ischemic heart disease or diabetes was mentioned on the certificate. As mentioned above, 77.1% of the individual with ischemic heart disease mentioned on their death certificate had it reported as the underlying. The second most common underlying cause of death when ischemic heart disease was mentioned was, in fact, diabetes (3.4% of the time underlying), followed by cerebrovascular disease (2.7% of the time underlying), then pneumonia (1.5% of the time underlying). When we focus on the population that has diabetes mentioned on the death certificate, as mentioned above 29.3% of the time diabetes is selected as the underlying, and the second most common underlying cause selected is ischemic hear disease at 25.4%, followed by cerebrovascular disease at 7.75% then followed by Other diseases of the heart 2.9%.

## Discussion

Distinct differences in the frequency of multiple causes of death were found across time, age, race, disposal method and place of death. Definitive explanations for the differences cannot be given based on this study, but it is of interest to consider plausible explanations which may motivate further investigation. The increased reporting of non-underlying causes of death as the age of the decedent increases is likely due to actual increases in co-morbidity with age, hence would be explained by actual differences in the causes of death.

The differences found in reporting of multiple causes of death for the other factors may be partly due to systematic reporting biases. According to the NCHS All Mortality Altas [[11], p. 3], the quality of cause of death determination in the US is affected by the accuracy and completeness of information – from medical diagnosis to final coding and processing of underlying cause of death. Although since 1968 the automated selection of the underlying cause of death has helped to reduce coding and processing errors, the completeness and accuracy of the information supplied on the certificate and the decedent's medical diagnosis remain as potential sources of error. If the certifier enters only one underlying cause and no other causes, then that cause will have to be selected as the underlying and there will not be multiple causes of deaths for that record. It is interesting to note that "Symptoms and ill-defined conditions" is the 5^th ^most commonly reported cause of death to be the only cause of death listed on the certificate. This reporting of it as the only cause of death pushes it up to be the overall 12^th ^leading cause of death. If almost any other cause would be listed simultaneously on the death certificate, this code would not end up as underlying.

The decreasing trend in reporting multiple causes over the decade may be reflective of a gradual change in the procedures of death certification. It would be of interest to consider this trend across different states and longer periods of time including shifts from one ICD coding system to the next.

Previous literature offers various plausible explanations to what contributes to the inaccuracy of reporting causes of death. The cause of death reported on the death certificates depends on a person's disease history that leads to death [12]. If a person dies after a long, well-characterized illness, the cause of death on the certificate is likely to be more accurate than a sudden or unobserved death. Also, when lack of adequate information on the decedent's disease history, the more narrowly characterized the cause of death on the certificate, the more likely it is to be in error. If we assume that reporting multiple causes on the death certificate can be considerd a proxy for level of familiarity of the death certifier with the patient, we would expect that a death which occurs in a hospital or nursing home would be more likely to have multiple causes reported, possibly due to a better documentation of disease history. On the other hand, death at the ER and in particular at the person's residence, which is conceivably often sudden should show a much lower percentage of multiple cause of death reporting. Analysis results from this current study match such speculations, supporting the argument that a good understanding of disease history is crucial. Still another result that supports this conclusion is the fact that performing autopsy, which gain better understanding of the disease condition, increased the probability of reporting multiple cause of death.

Gender and race can also play a role in the accuracy of reporting. Lloyd [13] showed that positive predictive value of the death certificate tended to be lower in women than in men. Although no large differences were seen between men and women with respect to frequency of multiple causes, there was a higher percentage of multiple causes reported for Native Americans. It is conceivable that the high percentage of multiple cause of death observed for Native Americans might be associated with the geographic factors of concentrated residence and the unique practices of local clinics. Moreover, results (not shown) indicate differences exist across counties of Minnesota in the reporting of multiple causes of death ranging from 50% to 80%. These results support suggestions for better standardized training for physicians and coroners.

Similar to the case of reporting multiple cause of death, the selection of ischemic heart disease and diabetes as underlying compared to non-underlying differs across the several factors considered. The implications of these differences across demographics are that mortality rates would be differentially affected when underlying cause of death is used compared to any mention cause of death. For example, for diabetes we might conclude that diabetes is being under-reported in Whites compared to Blacks, Native Americans and Hispanics if only underlying cause of death were considered since the proportion of diabetes as underlying to mentioned is substantially lower in Whites. This is not to say there is any inaccuracy in the way it is being coded but it points out where multiple cause of death reporting will provide a different perspective than underlying.

Nevertheless, studies have shown the sensitivity and positive predictive value of the death certificate are particularly poor with regard to stroke and diabetes [14]. Furthermore, Lloyd [13] concluded that physicians may use coronary heart disease as a "default" cause when facing some unknown cause of death cases. The fact that individuals with autopsy performed have lower probability of having heart disease selected as underlying when it is mentioned might suggest that heart disease is often over-assigned as the default disease when no further medical details are available. This is further demonstrated by the very high ratio of ischemic heart disease being coded as the underlying compared to non-underlying cause of death when the death occurred at the person's residence.

As mentioned in the introduction, one limitation of this research is the fact that there is no outside panel of experts who decide independently what the true causes of death are for each decedent, thus whether the associations we found are due to actual differences or reporting bias cannot be discerned. Therefore, this study cannot provide sensitivity or specificity per se, but it aims to identify factors that are associated with variability in reporting multiple cause of death and that perhaps contribute to inaccuracy in reporting underlying cause.

## Conclusions

There is much to be learned from multiple cause of death data. It provides ways of looking at mortality data that go well beyond the typical examination of underlying cause of death. Future research is needed to understand further what the greatest concerns are about the accuracy of reporting causes of death. Multiple cause of death data have the potential to help point out potential concerns in the accuracy as well as provide a more complete picture of mortality for causes which are frequently not recorded as the underlying cause of death.

## Competing interests

The author(s) declare that they have no competing interests.

## Authors' contributions

MW conceived of the study and wrote most of the manuscript. JH performed the statistical analysis and wrote part of the manuscript. JO provided access to the data and collaboration regarding processes underlying data collection. DD provided details about cause of death reporting and collaboration regarding processes underlying data collection. All authors read and approved the final manuscript.

## Pre-publication history

The pre-publication history for this paper can be accessed here:


